# Genetic characterisation of infectious bursal disease virus isolates in Ethiopia

**DOI:** 10.1016/j.actatropica.2013.09.025

**Published:** 2014-02

**Authors:** Shiferaw Jenberie, Stacey E. Lynch, Fekadu Kebede, Robert M. Christley, Esayas Gelaye, Haileleul Negussie, Kassahun Asmare, Gelagay Ayelet

**Affiliations:** a National Veterinary Institute, Debre-Zeit, Ethiopia; b The Institute of Infection and Global Health, University of Liverpool, Leahurst Campus, CH64 7TE, United Kingdom; c International Livestock Research Institute, Addis Ababa, Ethiopia; d College of Veterinary Medicine and Agriculture, Addis Ababa University, Debre-Zeit, Ethiopia; e Hawassa University School of Veterinary Medicine, P.O. Box 05, Hawassa, Ethiopia; f Norwegian School of Veterinary Science, Department of Food Safety and Infection Biology, Centre for Epidemiology and Biostatistics, P.O. Box 8146 Dep., 0033 Oslo, Norway

**Keywords:** Infectious bursal disease, vvIBDV, VP2, RT-PCR, Sequencing, Phylogenetic

## Abstract

The objective of the investigation was to characterise infectious bursal disease viruses (IBDV) circulating in commercial and breeding poultry farms in Ethiopia between 2009 and 2011. The nucleotide and deduced amino acid sequence for VP2 hypervariable region of ten IBDVs were determined by RT-PCR, sequenced and compared to well characterised IBDV isolates worldwide. IBDV genetic material was amplified directly from bursa or cell passaged material. Phylogenetically, Ethiopian IBDVs represented two genetic lineages: very virulent (vv) IBDVs or variants of the classical attenuated vaccine strain (D78). The nucleotide identity between Ethiopian vvIBDVs ranged between 0% and 2.6%. Ethiopian vvIBDVs are clustered phylogenetically with the African IBDV genetic lineage, independent of the Asian/European lineage. This report demonstrates the circulation of vvIBDV in commercial and breeding poultry farms in Ethiopia.

## Highlights

• We reported very virulent IBDV in Ethiopia.
• Ethiopian vvIBDVs cluster with vvIBDVs from Tanzania and Nigeria.
• Virulent IBDVs lacking the genetic markers of vvIBDV.

## Graphical abstract

This paper describes a molecular characterisation of IBD circulating in commercial and breeding poultry farms in Ethiopia. Ethiopian vvIBDVs are clustered phylogenetically with the African IBDV genetic lineage, independent of the Asian/European genetic.

## 1 Introduction

Infectious bursal disease virus (IBDV) belongs to the *Birnaviridae* family and has a non-enveloped, icosahedral capsid. The genome of virus is double-stranded RNA and bi-segmented (Eterradossi and Saif, 2008). Infectious bursal disease virus replicates in differentiating lymphocytes of the Bursa of Fabricius, causing the immunosuppressive and often fatal condition called infectious bursal disease (IBD) or Gumboro. IBDV consists of two serotypes, 1 and 2. Serotype 1 viruses are infectious for chickens, differing in their pathogenicity and are classified as avirulent, classical, variant and very virulent (vv) strains (Muller et al., 2003; Sapats and Ignjatovic, 2000).

Variant and vvIBDV strains have been isolated from disease outbreaks despite the presence of high levels of maternal antibody to classic strains of IBDV (Jackwood and Saif, 1987). The use of an appropriate vaccine is vital for effective protection and hence differentiation and identification of local IBDV isolates is crucial for selection of appropriate vaccine strain. Amplification of IBD virus protein 2 (VP2) gene and linking genetic variation found in this region with antigenic variation has been the major focus for strain identification in recent years (Bayliss et al., 1990; Brown et al., 1994; Wu et al., 2007). The IBDV VP2 hypervariable region (HVR) is commonly used to differentiate IBDV strains (Jackwood, 2004).

The first report of IBD in Ethiopia was in 2005 involving 20–45 day old broiler and layer chickens from commercial farms (Zeleke et al., 2005). Subsequently, IBD has become a priority problem in commercial and backyard poultry production system despite regular vaccination practices (in some cases) using attenuated IBDV D78 vaccine and improved biosecurity measures. This study was therefore initiated to determine the molecular characteristics of IBDV responsible for wide spread mortality and morbidity in Ethiopia.

## 2 Materials and methods

### 2.1 Study design

In this study, over 25 outbreaks of IBD were investigated by the National Veterinary Institute, Ethiopia, between 2009 and 2011. Outbreaks occurred in both commercial and breeding poultry farms under private and government ownership. This study involved the collection of clinical and epidemiological data, and the post-mortem examination of sick birds.

### 2.2 Bursa sample collection and virus isolation

For virus detection, bursa samples were aseptically collected from suspected cases, placed into individual sterile universal bottles and transported under cold chain to the virology laboratory, National Veterinary Institute, Ethiopia. Bursa samples were chopped into small pieces using a sterile scalpel blade, and minced using a mortar and pestle. A 10% suspension of each bursa sample was prepared in sterile phosphate buffer saline supplemented with penicillin and streptomycin (1000 μg/ml each). The suspension was transferred into sterile centrifuge tube and centrifuged at 3000 × *g* for 10 min. The supernatant was harvested and filtered using 0.22 μ milipore filters.

Samples of the resulting suspension were added to FTA card (Whatman) (to capture the viral RNA for molecular analysis) and inoculated onto confluent primary chicken fibroblast cell cultures for virus isolation. Cultures were maintained in GMEM containing 2% bovine calf serum and incubated at 37 °C. Cultures were observed microscopically for up to seven days for the presence of cytopathic effect (CPE) characteristic of IBDV. After seven days, samples with no CPE were blindly passed further three times following two cycles of freeze–thawing. Samples showing no CPE after the third passage were considered negative. Supernatant fluid from CPE positive cultures were also added onto FTA cards for molecular analysis. A total of ten IBDV specimens were available for genetic analysis, seven from original bursa material and three from virus isolated in cell culture.

### 2.3 RNA extraction and reverse transcription

RNA was eluted from FTA card by placing a section of the filter card (approximately 0.5 cm × 0.5 cm) in 300 μl of Elution buffer (Qiagen), vortexing and incubating on ice for approximately 15 min. Subsequently, 140 μl was further processed using the QIAGEN Viral RNA extraction kit as outlined by the manufacturer. Complementary DNA was generated from RNA using the reverse transcriptase RevertAid™ (Fermentas). RNA was first incubated at 95 °C for 3 min and placed on ice for at least 3 min in the presence of the gene specific primer L2 (5′-GATCCTGTTGCCACTCTTTC-3′), which binds nucleotides 1194–1213 of the positive strand of IBDV segment A (Bayliss et al., 1990), and 20% DMSO (Martin et al., 2007). RNA was reverse transcribed in a final volume of 20 μl containing reaction buffer (Fermentas), 1 mM of each dNTP (Thermo Scientific), 20 U RiboLock™ RNase Inhibitor and 200 units RevertAid™ Reverse transcriptase. Reverse transcription reactions were performed at 42 °C for 60 min and the reverse transcriptase inactivated at 70 °C for 10 min.

### 2.4 PCR amplification and sequencing

Polymerase chain reaction (PCR) amplification of products intended for sequencing was carried out using a high fidelity DNA polymerase, *Pfu* DNA polymerase (Fermentas). A typical 25 μl reaction contained *Pfu* Buffer with MgSO_4_ (Fermentas), 0.2 mM dNTPs (Thermo Scientific), 200 nM of each primers L2 (5′-GATCCTGTTGCCACTCTTTC-3′) and U2 (5′-GGTATGTGAGGCTTGGTGAC-3′) which binds nucleotide position 1194–1213 and 658–677 of IBDV segment A, respectively (Bayliss et al., 1990), 2.5 units (U) *Pfu*DNA polymerase (Fermentas) and 2 μl of cDNA template. PCR reactions were carried out for 1 cycle at 95 °C for 3 min, 35 cycles at 95 °C for 30 s, 60 °C for 30 s, 72 °C for 1 min and 1 cycle at 72 °C for 7 min. The amplified 604 base pair product contained the VP2 hypervariable region coding sequence. Amplicons were separated from reaction components using the QIAGEN Gel extraction kit, with the concentration of DNA determined spectrophotometrically using a Nano Drop Spectrophometer 1000 (Thermo Scientific). Purified amplicons were sequenced using both L2 and U2 primers by a commercial sequence provider (Macrogen) using the Big Dye terminator cycling (Applied Biosystems) condition and analysed by the automated sequencer ABI 3730XL.

### 2.5 Sequence and Phylogenetic analysis

Sequences were analyses using Geneious (Drummond et al., 2011). The deduced amino acid sequence (amino acid position 191–350) of Ethiopian IBDV isolates sequenced in this study were alignment to the VP2 amino acid 239–332 (Bayliss et al., 1990) of well characterised IBDVs. Phylogenetic analysis was based on an alignment of partial IBDV VP2 sequences (nucleotide position 804–1190), spanning the HVR at nucleotide position 845–1126 (Bayliss et al., 1990). Nucleotide alignment was performed using ClustalW, within Mega 5.1 (Tamura et al., 2011), with the phylogenetic analysis inferred using the neighbour-joining method. One thousand bootstrapping replicates were used to estimate the robustness of tree branches.

## 3 Results

### 3.1 Nucleotide and deduced amino acid sequence

The nucleotide sequence of the VP2 HVR was determined for 10 Ethiopian IBDV isolates from cDNA transcripts. Nucleotide identity between the 10 isolates ranged between 90.2% and 100%. Isolates IBDV 15/10 and IBDV 16/10 showed 100% identity over the region sequenced and were genetically related to IBDV 11/10, 6/10, 17/10 and 01/10 (nucleotide identity 99.8%). Isolates IBDV 03/11, 04/09 and 10/10 showed 100% nucleotide identity over the region sequenced and were genetically related to IBDV 09/09 (97.7%). Nucleotide identity between the Ethiopian produced IBDV vaccine and Ethiopian IBDV isolates ranged between 90.2% and 94.6%.

**Fig. 1:**
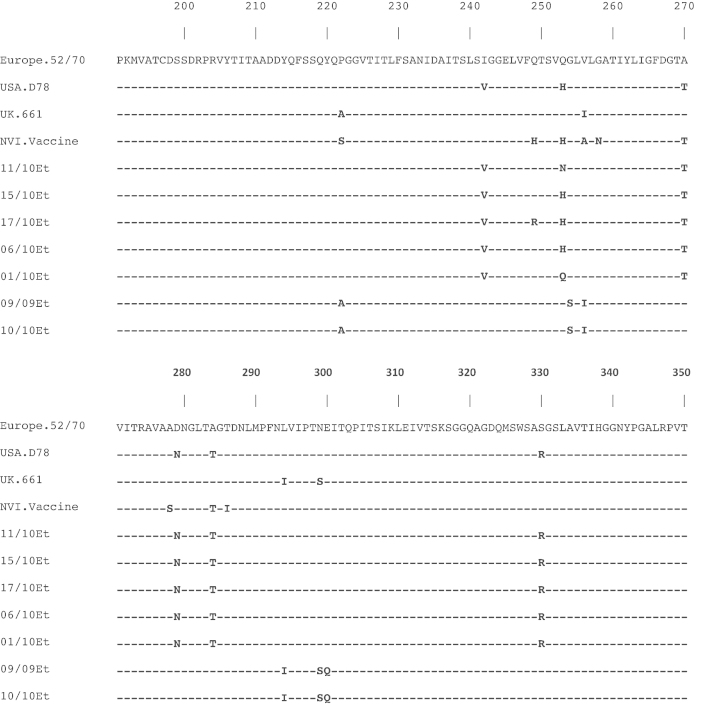
Deduced amino acid alignment of VP2 hypervariable region of Ethiopian IBDV isolates sequenced in this study (Table 1); classical IBDV (Europe.F52/70, Y14958); classical attenuation IBDV (USA.D78, EU162087); vvIBDV (UK.661, Z25480) and an Ethiopian produced IBDV vaccine (NVI Vaccine) (Table 1). Dashes indicate amino acids identical to Europe.F52/70.

The deduced amino acid sequence of the hypervariable region was determined for each of the isolates and compared to well characterise classical virulent IBDV isolates (F52/70), classical attenuated IBDV isolates (IBDV 78) and vvIBDV isolates (vvIBDV UK 661) (Fig. 1). Four Ethiopian IBDV isolates (IBDV 03/11, 04/09, 09/09 and 10/10) contain the genetic signature of vvIBDVs, specifically, A222, I256, I294, S299 (Brown et al., 1994), however, lacked L324 and V321, characteristic of antigenically atypical vvIBDV (Eterradossi et al., 1998). Six of the IBDV isolates (IBDV 12/10, 15/10, 11/10, 06/10, 17/10 and 16/10) contain amino acid sequences linked to propagation in cell culture (N279 and T284) (Lim et al., 1999; Mundt, 1999). Interestingly, heterogeneous variation was also detected in these six isolates at amino acid 253 (H/Q/N), a residue shown to be involved in both cell tropism and virulence (Boot et al., 2000; Brandt et al., 2001; Qi et al., 2009; Van Loon et al., 2002).

### 3.2 Phlyogenetic analysis

**Fig. 2:**
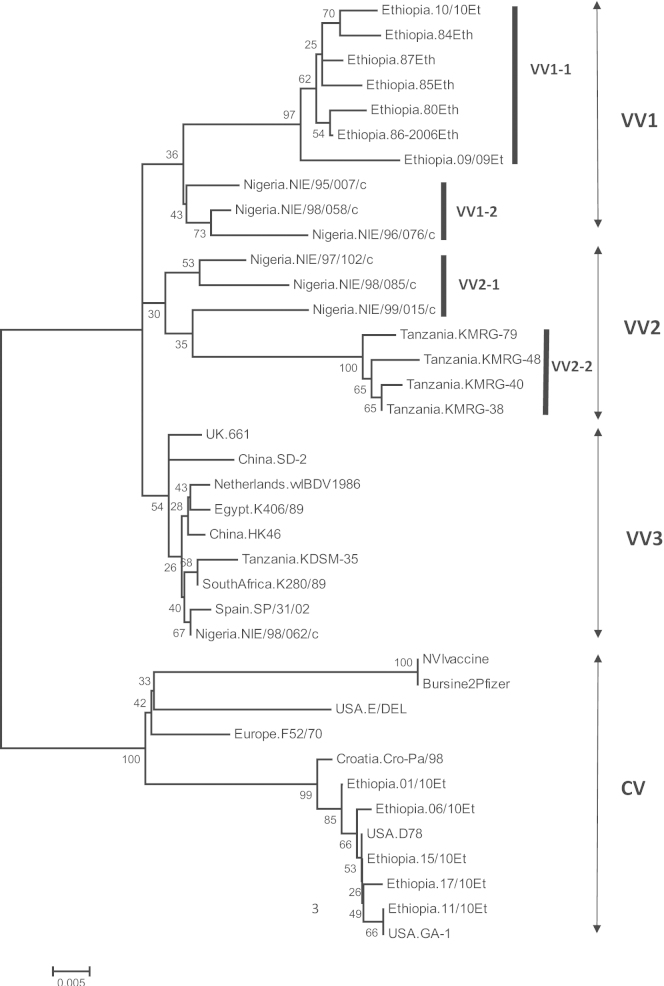
Phylogenetic analysis of the VP2 hypervariable coding sequence of 38 IBDV isolates. The neighbour-joining consensus tree is shown. Results of the 1000 bootstrapping replicates are represented above or below forks as a percentage. Infectious bursal disease isolates group phylogenetically into Very Virulent (VV) subgroup VV1 (VV1-1 and VV1-2) and subgroup VV2 (VV2-1 and VV2-2) and classical virulent strains (CV). Published sequenced used include: VV1-1 Ethiopia.80Eth (JF826458); Ethiopia.84Eth (JF826456); Ethiopia.87Eth (JF826454); Ethiopia.85Eth (JF826457); Ethiopia.86-2006Eth (HQ231797); VV1-2 Nigeria.NIE/98/058/c (AJ586951); Nigeria.NIE/95/007/c (AJ586917); Nigeria.NIE/96/076/c (AJ586924); VV2-1 Nigeria.NIE/97/102/c (AJ586939); Nigeria.NIE/98/085/c (AJ586945); Nigeria.NIE/99/015/c (AJ586955); VV2-2 Tanzania.KMRG-40 (AB200982); Tanzania.KMRG-79 (AB200986); Tanzania.KMRG-38 (AB200981); Tanzania.KMRG-48 (AB200983); VV3 Tanzania.KDSM-35 (AB200980); UK.661 (Z25480); Spain.SP/31/02 (AY770593); Egypt.K406/89 (AF159218); SouthAfrica.K280/89 (AF159217); China.HK46 (AF051838); Nigeria.NIE/98/062/c (AJ586946); China.SD-2 (EU042147); Netherlands.vvIBDV1986 (Z25482) and CV USA.GA-1 (EF418034); USA.D78 (EU162087); Croatia.Cro-Pa/98 (EU184689); Europe.F52/70 (Y14958); Bursine2Pfizer (AF498631); and USA.E/Del (X54858). Refer to Table 1 for accession numbers of the Ethiopian IBDV isolates sequenced as part of this study and grouped into VV1 and CV. The bar represents 0.005 nucleotide substitutions per site.

The VP2 HVR nucleotide sequence of Ethiopian isolates detected in this study were used in a nucleotide database search BLAST [http://blast.ncbi.nlm.nih.gov/Blast.cgi] to identify IBDV isolates with highest identity. The phenotypic relationship between IBDV isolates showing high nucleotide identity to the Ethiopian IBDVs sequenced in this study, in addition to several well characterised vvIBDVs and Classical IBDVs (inc. variant strains and attenuated vaccine strains) was inferred using the Neighbour-joining method (Fig. 2). For simplicity, the four Ethiopian IBDVs with 100% nucleotide identity to another Ethiopian IBDV were excluded from the figure. The Ethiopian IBDV isolates sequenced in this study represent two distinct genetic lineages (1) vvIBDV and (2) classical IBDV strains. Ethiopian isolates IBDV 10/10 and IBDV 09/09 were most closely related to vvIBDV isolates from Nigeria and unpublished Ethiopian IBDV isolates (genetic diversity 0.76–2.29%). Five of the Ethiopian isolates (IBDV 15/10, 11/10, 06/10, 17/10) clustered together with the classical attenuated vaccines strain D78.

### 3.3 Investigation into the genetic relatedness of Ethiopian IBDV isolates and IBDV D78

The genetic relatedness of several Ethiopian isolates (IBDV 11/10, 6/10, 17/10, 01/10, 16/10 and 15/10) and a classical attenuated vaccine strain D78 was further examined by comparing the nucleotide and deduced amino acid sequence of VP2 HVR. The nucleotide sequences of two isolates (IBDV 15/10 and 16/10) were 100% identical to a published sequence of D78. A single point mutation, resulting in a single amino acid change, was detected in three Ethiopian IBDV isolates: IBDV 11/10, a nucleotide change at position 888 (C → A) resulted in an asparagine (N) at position 253; IBDV 17/10, a nucleotide change at position 877 (A → G) resulted in a arginine (R) at position 249 and IBDV 01/10, a nucleotide change at position 890 (C → A) resulted in a glutamine (Q) at position 253. A silent point mutation was detected in IBDV 06/10 at position 965 (A → G).

## 4 Discussion

This study demonstrated that vvIBDV genotype is circulating in Ethiopia. The amino acid characteristic of vvIBDVs (222A, 256I, 294I and 299S) (Brown et al., 1994) were detected in the vvIBDVs analysed. Phylogenetic analyses of these and other unpublished Ethiopian IBDV sequences demonstrate the clustering of Ethiopian vvIBDVs within the tentatively named African VV1 lineage (Kasanga et al., 2007), independent of the Asian/European lineage (VV3). Although Ethiopian and Nigerian vvIBDVs share a common ancestor, VP2 HVR sequences group into two distinct clusters (tentatively named VV1-1 and VV1-2) within VV1. Sequence divergence from a single origin within multiple countries is consistent with the proposed evolution of the two distinct clusters in the African VV2 lineage, specifically, VV2-1 (Nigerian vvIBDVs) and VV2-2 (Tanzania vvIBDVs) (Kasanga et al., 2007). Investigations into the molecular epidemiology of vvIBDV in other African countries (such as Uganda, Sudan, and Kenya) where their VP2 HVR sequence data is not publically available, may provide further phylogenetic insight into the origin and molecular epidemiology of vvIBDVs.

Ethiopian vvIBDVs show considerable genetic homogeneity (0.0–2.6%), with phylogenetic analysis suggesting a single origin. This is in contrast to the sequence heterogeneity detected among vvIBDV isolates from other African countries. In Nigeria, vvIBDVs show a high level of genetic heterogeneity (5.7%) and two distinct genetic clusters (specifically VV1 and VV2-1) (Owoade et al., 2004), while there is evidence of vvIBDV isolates from both Asian/European lineages circulating in Tanzania (Kasanga et al., 2007) and Nigeria (Zierenberg et al., 2000). Three of the four vvIBDV isolates (IBDV 03/11, 04/09 and 10/10) sequenced as part of this study had 100% nucleotide identity in VP2 HVR, despite being isolated over two years. Genetic stability over time within the VP2 HVR has been detected in Nigeria (Owoade et al., 2004) and Italy (Martin et al., 2007), and thought to be maintained by low immune pressure or presence of a non replicative, static virus source (Owoade et al., 2004).

Six of the 10 Ethiopian IBDV isolates sequenced as part of this study were phylogenetically related to classical attenuated vaccine IBDV D78, despite being detected from bursae with significant gross pathology of the bursa. Virulent genetic variants of IBDV D78 have been documented following only a single point mutation at position 253 (GA-1)(Brandt et al., 2001; Jackwood et al., 2008), or the emergence of a virulent genetic variant within the heterogeneous genetic population present in some commercial IBDV vaccines (Jackwood and Sommer, 2002). Virulent genetic variants of the IBDV D78 vaccine strain, such as GA-1, have been isolated from vaccinated flocks when the manufacturers’ recommendations have not been followed (e.g. administration at a reduced dosage) (Jackwood et al., 2008). The VP2 HVR of IBDV 11/10 was 100% identical to GA-1. Single amino acid substitutions in the VP2 HVR at position 253 (N or Q) have experimentally been linked to increased virulence in vivo (Jackwood et al., 2008). Interestingly, heterogeneous variation at this position was also detected in this study (IBDV 11/10 (N253) and IBDV 11/10 (Q253)). It would therefore be interesting to examine if like earlier studies, amino acid variation at this position is linked with virulence. Virulent IBDV genetically related to IBDV D78, have also been reported in Italy, Croatia and Canada (Lojkic et al., 2008; Martin et al., 2007; Ojkic et al., 2007).

The deduced amino acid sequence of the remaining Ethiopian IBDVs (IBDV 15/10, 16/10 and 17/10) was 100% identical to D78. Analysis of these isolates is difficult as cell culture material was used for the genetic analysis and in vitro passaging can affect the nucleotide sequence of VP2 HVR (Brandt et al., 2001) and leads to reduced genetic diversity (population bottlenecking).

Although classical attenuated IBDV vaccines protect chickens from vvIBDV, a hallmark of vvIBDVs is the ability to cause disease in the presence of higher levels of neutralising antibodies in comparison to the classical virulent IBDVs. Therefore, highly attenuated vaccines that induce lower levels of neutralising antibody, such as Bursine 2, may not provide adequate vaccination induced protection against vvIBDVs. Furthermore, as variations in the deduced amino acid sequence at key neutralisation epitopes (amino acid position 222 (Eterradossi et al., 1998) exists between the vaccines currently distributed in Ethiopia (NVI vaccine and D78) and Ethiopian vvIBDVs, alternative vaccine strains may prove more efficacious. Vaccination with cell culture attenuated vvIBDV have been found efficacious (Rasool and Hussain, 2006), however, the risk for reversion to virulence (Raue et al., 2004) may hinder commercialisation. A driver for reversion to virulence of attenuated vaccine strains is the inappropriate usage of vaccines (i.e. lower dose) (Raue et al., 2004). In this and other studies (Jackwood et al., 2008; Lojkic et al., 2008; Martin et al., 2007; Ojkic et al., 2007), virulent classical viruses, genetically related to current attenuated classical vaccine strains, are responsible for outbreaks. Therefore, ensuring appropriate vaccine usage should be adequately addressed before attenuated vvIBDVs are used for vaccine production.

In this study we have reported IBDV with the genetic markers of typical vvIBDVs circulating in Ethiopia. Ethiopian vvIBDVs cluster phylogenetically with vvIBDVs from Tanzania and Nigeria in an African genetic lineage, independent of the Asian/European genetic lineage. Virulent IBDVs lacking the genetic markers of vvIBDV, however, genetically related to the attenuated classical vaccine strains, D78 were also detected. Information from this study could be used to guide IBDV vaccine selection in Ethiopia and further supports an independent vvIBDV African lineage.

## Figures and Tables

**Table 1:** Description of IBDV isolates included in this study.

Virus isolate	Date of collection	Sample type	Age of bird (days)	GenBank accession no.	Phylogenetic group
IBDV 01/10	12/02/10	Bursa	7	JQ684021	CV
IBDV 03/11	14/07/11	Bursa	31	See JQ68401	VV
IBDV 04/09	07/09/09	Bursa	30	See JQ68401	VV
IBDV 06/10	28/04/10	Bursa	31	JQ684020	CV
IBDV 10/10	09/12/10	Bursa	85	JQ68401	VV
IBDV 11/10	8/09/10	Bursa	29	JQ684016	CV
IBDV 17/10	04/05/11	Cell culture (p10^b^)	N/A	JQ684017	CV
IBDV 16/10	27/07/10	Cell culture (p7)	N/A	See JQ684018	CV
IBDV 15/10 t	31/12/10	Cell culture (p1)	N/A	JQ684018	CV
IBDV 09/09	24/08/09	Bursa	30	JQ684019	VV
NVI vaccine	N/A	N/A	N/A	JQ684022	CV

N/A – not applicable. ^a^Very virulent (VV), classical virulent (CV).

b Passage number.
